# 2D Rotation-Angle Measurement Utilizing Least Iterative Region Segmentation

**DOI:** 10.3390/s19071634

**Published:** 2019-04-05

**Authors:** Chenguang Cao, Qi Ouyang

**Affiliations:** School of Automation, Chongqing University, Chongqing 400044, China; guangcc@foxmail.com

**Keywords:** geometric moments, camera pose, rotation-angle, measurement error

## Abstract

When geometric moments are used to measure the rotation-angle of plane workpieces, the same rotation angle would be obtained with dissimilar poses. Such a case would be shown as an error in an automatic sorting system. Here, we present an improved rotation-angle measurement method based on geometric moments, which is suitable for automatic sorting systems. The method can overcome this limitation to obtain accurate results. The accuracy, speed, and generality of this method are analyzed in detail. In addition, a rotation-angle measurement error model is established to study the effect of camera pose on the rotation-angle measurement accuracy. We find that a rotation-angle measurement error will occur with a non-ideal camera pose. Thus, a correction method is proposed to increase accuracy and reduce the measurement error caused by camera pose. Finally, an automatic sorting system is developed, and experiments are conducted to verify the effectiveness of our methods. The experimental results show that the rotation angles are accurately obtained and workpieces could be correctly placed by this system.

## 1. Introduction

An automatic sorting system has the advantages of high efficiency, low error rate, and low labor cost. It is widely used in several fields, such as vegetable classification [1,2], the postal industry [3], waste recycling [4,5], and medicine [6]. To meet the requirements of intelligent sorting in industrial environments, a vision system is often used to sense, observe, and control the sorting process. In this system, the pose and position of the workpiece are obtained using a camera with image processing, and the actuator is driven according to these parameters. Consequently, the adaptive ability of the automatic sorting system will be improved. Generally, pose is described by angles in space [7]. For a plane workpiece, only one angle is needed. To place a plane workpiece correctly, the rotation angle of each workpiece must be calculated. Because workpieces are placed arbitrarily in the sorting area, they should be placed in the storage area in one pose. Therefore, the rotation-angle is an important parameter that is used to plan a path for the actuator. An incorrect rotation-angle will lead to an error in path planning, causing the workpieces to be placed incorrectly.

Rotation-angle measurement is an important component of visual measurement and has been substantially studied. As a result, various visual rotation-angle measurement methods have emerged, and they are used in different fields. Existing rotation-angle measurement methods are mainly classified into four categories. The first one is template matching, in which the rotation angle is calculated through a similarity measurement. This method is simple, but it has a high computational cost and is slow. The main challenges for template matching are the reduction of its computational cost and improvement of its efficiency [8]. The second category is polar transform. The advantage of this method is that any rotation and scale in Cartesian coordinates are represented as shifts in the angular and the log-radius directions in log-polar coordinates, respectively [9]. Then, the rotation angle is obtained between two images. The third category of methods takes advantage of the feature line and feature point in images. The feature line is obtained by Hough transformation or from the image moment [10]. The angle between the matching lines in two images is calculated, and it could be regarded as the rotation angle. Such methods are simple and suitable for fast detection. Feature points are some local features that are extracted from the image. They remain constant for rotation, scaling, and various types of illumination. Scale-invariant feature transform (SIFT) is always used to obtain feature points [11], and the rotation angle is calculated by matching points in different images. In [12], a isosceles triangle is established and then the rotation-angle between two points could be obtained by solving the triangle formed by origin coordinates and position of these two points. The fourth category of methods requires auxiliary equipment, which mainly include a calibration board and projector. In [13], a calibration pattern with a spot array was installed at the rotor. The rotation angle of the spot array is detected with the equation of coordinate rotation measurement. The standard deviation of rotation-angle measurement is smaller than 3 arcsec. In [14], a practical method to measure single-axis rotation angles with conveniently acquirable equipment was presented. Experiments achieved a measurement accuracy of less than $0.1^{\circ }$ with a camera and a printed checkboard. The Moire fringe is an optical phenomenon used in rotation-angle measurement. The principle of measurement is that the width of the Moire fringe varies as the angle between the grating lines varies [15]. Lensless digital holographic microscopy is used to accurately measure ultrasmall rotation angles [16]. Furthermore, white-light interferometry was used in a previous study to measure one-dimensional rotation angles [17]. In that study, the rotation angle was measured with an optical plane-parallel plate with a standard refractive index. The phase change of the interference spectrum of the interferometer was output during the rotation of the plane workpiece.

It should be noted that although many methods have been developed, these methods are not suitable for automatic sorting system. This is because the method used in the automatic sorting system has three requirements. Firstly, the rotation angle needs to be calculated correctly in a short time. Secondly, auxiliary equipment should not be used, because of the continuous movement of the workpieces. Thirdly, the method has generality and can calculate the rotation-angle of different workpieces. Therefore, a new rotation-angle measurement method which satisfies the above conditions is needed.

In the present paper, an improved rotation-angle measurement method based on geometric moments is proposed. The improved method is suitable for workpieces of all shapes and could overcome a limitation of geometric moments when calculating the rotation-angle. The analysis of speed and accuracy of the proposed method shows that it can meet the requirements of automatic sorting systems. In addition, a rotation-angle measurement model is established, and the relationship between camera pose and rotation-angle measurement error is investigated. Subsequently, a correction method is presented to reduce the measurement error caused by camera pose. Experimental results show that this method is accurate and suitable for rotation-angle measurement. The remainder of this paper is organized as follows. Section 2 reviews the concept of image moment and clarifies the limitation of rotation-angle measurement based on geometric moments. Section 3 describes the rotation-angle measurement method in detail. Section 4 establishes a rotation-angle measurement model and illustrates that a measurement error can be caused by camera pose. Subsequently, a correction method for rotation-angle measurement error is presented. In Section 5, an automatic sorting system is set up, and experimental results are discussed. Section 6 draws conclusions.

## 2. Basic Theory

### 2.1. Image Moment

The concept of moments was initially proposed in classical mechanics and statistics. At present, it is widely used in image recognition [18,19], image segmentation [20], and digital compression [21]. The geometric moment of an image is the simplest, and lower moments have a clear physical meaning in an image.

Area is expressed by the zeroth moment:

(1) $$M_{00}=\sum_{i=1}^{n}\sum_{j=1}^{m}I\left(i,j\right).$$

Center of mass is expressed by the first moment:

(2) $$\left\{\begin{array}{c} M_{10}=\sum_{i=1}^{n}\sum_{j=1}^{m}i\cdot I\left(i,j\right)\\ M_{01}=\sum_{i=1}^{n}\sum_{j=1}^{m}j\cdot I\left(i,j\right) \end{array}\right.$$

(3) $$x_{c}=\frac{M_{10}}{M_{00}},y_{c}=\frac{M_{01}}{M_{00}}.$$

The second moments are defined as:

(4) $$\left\{\begin{array}{c} M_{20}=\sum_{i=1}^{n}\sum_{j=1}^{m}i^{2}\cdot I\left(i,j\right)\\ M_{02}=\sum_{i=1}^{n}\sum_{j=1}^{m}i\cdot j\cdot I\left(i,j\right)\\ M_{11}=\sum_{i=1}^{n}\sum_{j=1}^{m}j^{2}\cdot I\left(i,j\right) \end{array}\right.$$

Orientation is used to describe how the object lies in the field of view and it could be expressed by this three second moments:

(5) $$\tan2\theta =\frac{b}{a-c}$$

where

(6) $$\left\{\begin{array}{c} a=\frac{M_{20}}{M_{00}}-x_{c}^{2}\\ b=\frac{M_{11}}{M_{00}}-x_{c}y_{c}\\ c=\frac{M_{02}}{M_{00}}-y_{c}^{2} \end{array}\right.$$

θ is an angle which is defined by the direction of the axis of least inertia [22]. It is worth noting that the summation is used in Equations (1), (2) and (4) because we are dealing with discrete images rather than continuous images.

Higher moments contain details of the image that are relatively more sensitive to noise. Redundancies will be shown in the operation of a higher moment owing to its nonorthogonality. Therefore, many new moments [18,21,23] have been proposed.

### 2.2. Deficiency of Rotation-Angle Measurement Based on Geometric Moments

**Figure 1:**
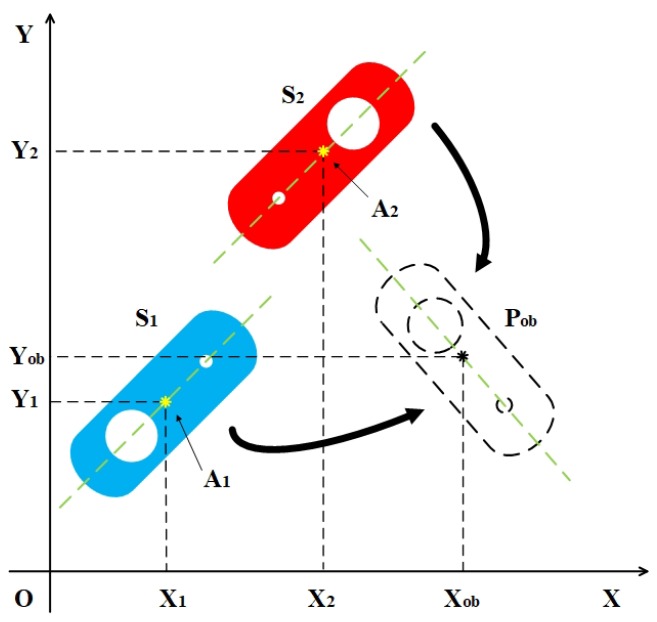
Case where the same rotation angle is obtained with dissimilar poses when using image geometric moments.

The rotation-angle measurement method based on geometric moments is advantageous because of its high accuracy and speed. However, there is a limitation when this method is used, as illustrated in Figure 1. The figure shows two workpieces $S_{1}$ and $S_{2}$ with different poses. Their centers of mass $A_{1}$ and $A_{2}$ are represented by the yellow ∗ symbols, and their axes are represented by green dotted lines. The angles $\theta_{1}$ and $\theta_{2}$ of the two axes are equal. The object position and pose are expressed by $P_{ob}$, and the angle of its axis is $\theta_{ob}$.

Assuming that the rotation direction is counterclockwise, when $S_{1}$ and $S_{2}$ need to be placed into $P_{ob}$, the minimum rotating angle around the center of mass is obtained as follows:

(7) $$r_{n}=\left\{\begin{array}{l} \theta_{ob}-\theta_{n}+180^{\circ },n=1.\\ \theta_{ob}-\theta_{n},n=2. \end{array}\right.$$

where *r* is the rotation angle.

The difference between $r_{1}$ and $r_{2}$ is $180^{\circ }$ because $\theta_{1}$ is equal to $\theta_{2}$. $S_{1}$ and $S_{2}$ will be rotated by the same angle if the angle of the axis is regarded as the rotation angle. Therefore, the same rotation angle is obtained with dissimilar poses, which is an error of the measurement. The reason is that $S_{1}$ and $S_{2}$ are non-centrosymmetric about points $A_{1}$ and $A_{2}$, respectively. Thus, for non-centrosymmetric workpieces, the same rotation-angle would be obtained with dissimilar poses when the geometric moments are used for rotation-angle measurement. An automatic sorting system using this method is only suitable for center-symmetrical workpieces. This limitation significantly decreases the generality of the system.

## 3. Method for Rotation-Angle Measurement

An improved method is presented here to overcome the limitation described in the previous section. The method is called the least iterative region segmentation (LIRS) method which consists of three steps and geometric information is used to overcome the limitation caused by the shape of the workpiece. The following two points are made before the LIRS method is introduced:

(1) We assume that the plane workpiece is uniform and the center of mass is located on the workpiece.
(2) We assume that the optical axis of the camera is perpendicular to the work plane.

The LIRS method is illustrated in detail below.

### 3.1. Image Preprocessing

An image point will be deviated from its ideal position in the presence of lens distortion [24], resulting in distorted images. Therefore, the calibration is used to improve the accuracy of rotation-angle measurement [25]. Moreover, the complicated background and the surface texture of a workpiece will appear as noise in rotation-angle measurement. Therefore, image processing is required to acquire a superior binary image. Common methods for image processing include denoising, grayscale, image morphology, and binarization. The image needs to be segmented into several pieces when more than one workpiece exists because only one workpiece can be handled at a time.

### 3.2. Least Iterative Region Segmentation Method

**Figure 2:**
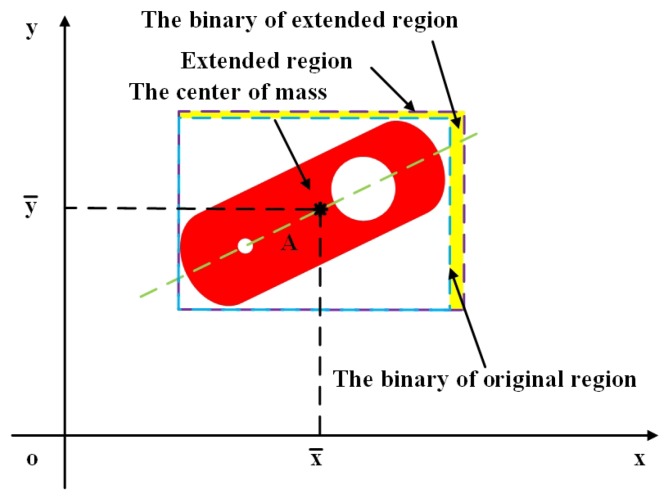
Schematic of image extension.

The coordinates system is established as shown in Figure 2. The red region is a workpiece. $I_{sw}$ is a regions which is the minimum enclosing rectangle of the workpiece. $I_{sw}^{\prime }$ is also a regions which boundary is violet dotted line. The axis is shown as blue dotted line and the angle of the axis θ is obtained from Equation (5). The point A is the center of mass which coordinate is $\left(\bar{x},\bar{y}\right)$.

#### 3.2.1. Judgment of Centrosymmetry

Two steps are required to judge whether the workpiece is centrosymmetric. Firstly, the center of $I_{sw}$ should be calculated and the region $I_{sw}$ needs to be extended to $I_{sw}^{\prime }$, if *A* is not the center of $I_{sd}$. After extention, the point *A* is the center of $I_{sd}^{\prime }$. Secondly, the region $I_{sd}^{\prime }$ rotated by $180^{\circ }$ is convolved with the original region. The workpiece is centrosymmetric about the center of mass if the result is greater than a threshold. The angle in the counterclockwise direction between the two axes can be regarded as the rotation angle. Otherwise, the next step should be carried out.

Template matching is always used for recognition. Therefore, this step can be changed to evaluate whether the template is centrosymmetric. The next step will be performed when the workpiece matches the asymmetric template. In this manner, the judgment of centrosymmetry will be completed before the region segmentation, and the efficiency of LIRS will be improved.

#### 3.2.2. Region Segmentation and Identification

**Figure 3:**
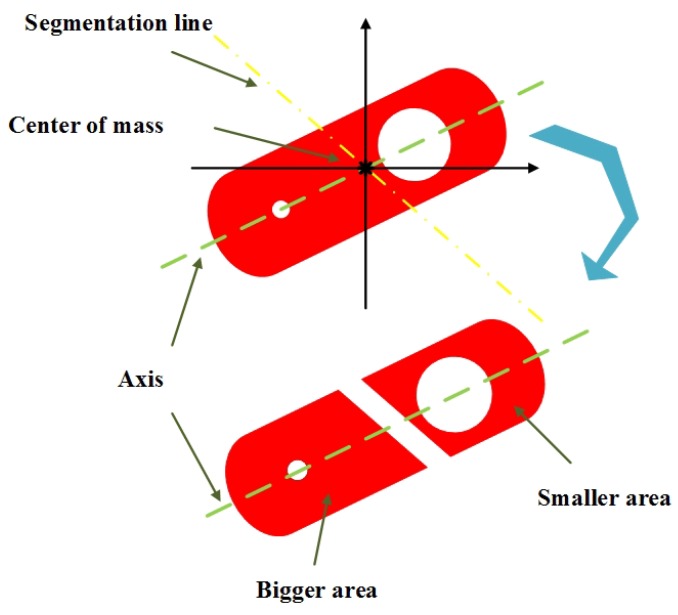
Image segmentation with a separation line.

The purpose of this subsection is to find a separation line which divide the workpiece into two parts with different areas. A new rectangular coordinate system is established with center of mass as its origin, as shown in Figure 3. Then, a separation line through the origin is drawn as follows:

(8) y-kx=0,

where k=tan(θ+nα), α is the deviation angle which range is $\left[0^{\circ },360^{\circ }\right)$ and *n* is the iteration number which initial value is 1.

After θ and *n* are assigned, the equation of the separation line would be obtained. Then the workpiece could be divided into two parts $D_{1}$ and $D_{2}$ according to the relationship between the point and the line. The areas of $D_{1}$ and $D_{2}$ are $\Gamma (D_{1})$ and $\Gamma (D_{2})$. When the $\Gamma (D_{1})$ is equal $\Gamma (D_{2})$, we need to add 1 to *n* and divide the workpiece with the new separation line. The iteration will be stopped until the condition $\Gamma \left(D_{1}\right)\neq \Gamma \left(D_{2}\right)$ is met. The larger between the two parts is marked as $D_{l}$ while the other is marked as $D_{s}$. The workpiece must be divided into two regions with different areas by the separation line because it is non-centrosymmetric about the center of mass.

To improve the efficiency of division, the threshold method is used. Firstly, the threshold function $B\left(P\right)=y-kx$ is established and $P\left(x,y\right)$ is a point in the workpiece. The segmentation function is set up as expressed by Equation (9), and $P\left(x,y\right)$ can be assigned to a region according to the polarity of the thresh function. Therefore, the workpiece is divided into two parts according to the relationship between the point and the separation line.

(9) $$\left\{\begin{array}{c} B\left(P\right)>0,P\in D_{1}\\ B\left(P\right)>0,P\in D_{2} \end{array}\right.$$

There are two point which need attention:

(1) The deviation angle needs to be selected reasonably. We should avoid choosing the symmetry axis or its perpendicular axis as the separation line because these axes divide a symmetric workpiece into two parts with the same area.
(2) The area of the workpiece will not be exactly equal after the workpiece is rotated at different angles because the images captured by the industrial camera have been already discretized by a charge-coupled device and a discretization error will always exist. To eliminate the effect of discretization on the measurement, a threshold is employed. The areas of $D_{l}$ and $D_{s}$ are considered equal when the absolute area difference is less than the threshold.

#### 3.2.3. Rotation-Angle Calculation

After segmentation, a direction vector $\vec{p}$ can be established from $\left(\bar{x_{l}},\bar{y_{l}}\right)$ to $\left(\bar{x_{s}},\bar{y_{s}}\right)$, where $\left(\bar{x_{l}},\bar{y_{l}}\right)$ is the center of mass of $D_{l}$ and $\left(\bar{x_{s}},\bar{y_{s}}\right)$ is the center of mass of $D_{s}$. The two coordinates are calculated by Equation (3). The direction vector can be used to calculate the rotation angle because of rotation invariance. Assuming that a pose is represented by vector $\vec{p}=\left(x_{o},y_{o}\right)$, the rotation angle is obtained by employing:

(10) $$\begin{array}{c} \Theta =\frac{\vec{p}\times \vec{q}}{|\vec{p}\left|\right|\vec{q}|}=\frac{\Delta xx_{o}+\Delta yy_{0}}{\sqrt{{\left(\Delta x\right)}^{2}+{\left(\Delta y\right)}^{2}}\sqrt{x_{o}^{2}+y_{o}^{2}}}\\ \Delta x=x_{s}-x_{l},\Delta y=y_{s}-y_{l} \end{array}$$

(11) $$\Lambda =\vec{p}\times \vec{q}$$

(12) $$\theta =f\left(\Theta ,\Lambda \right)$$

where, Θ is a cosine value and the Λ is symbol which polarity is decided by the relationship between $\vec{q}$ and $\vec{p}$. *f* is a function which calculate the rotation-angle based on Θ and the Λ. The value range of θ is $\left[0^{\circ },360^{\circ }\right)$.

**Figure 4:**
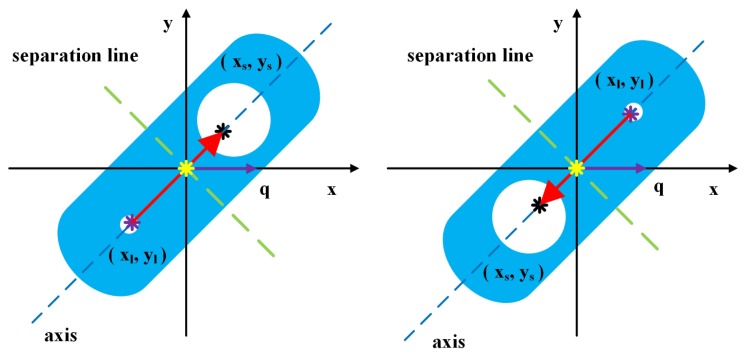
Result of the LIRS method.

The result of the LIRS method is shown in Figure 4. The green dotted lines are the separation lines and the blue dotted lines are axes. The red arrows are the direction vectors and the purple arrows are the object vectors. Although the slopes of the two axes are the same, the direction vectors are different. The angle in the counterclockwise direction between two direction vectors could be regarded as the rotation-angle. The result shows that the LIRS method can effectively measure the rotation-angle of the workpiece, and it overcomes the limitation of the conventional rotation-angle measurement method based on geometric moments.

### 3.3. Evaluation of LIRS Method

Efficiency, accuracy, and application range are the three most important indexes of automatic sorting systems. They are affected by the performance of the vision algorithm. Therefore, the applicability of the LIRS method in industrial environments needs to be evaluated. In this section, the accuracy, speed, and generality of LIRS as well as the image size are analyzed in detail.

**Figure 5:**
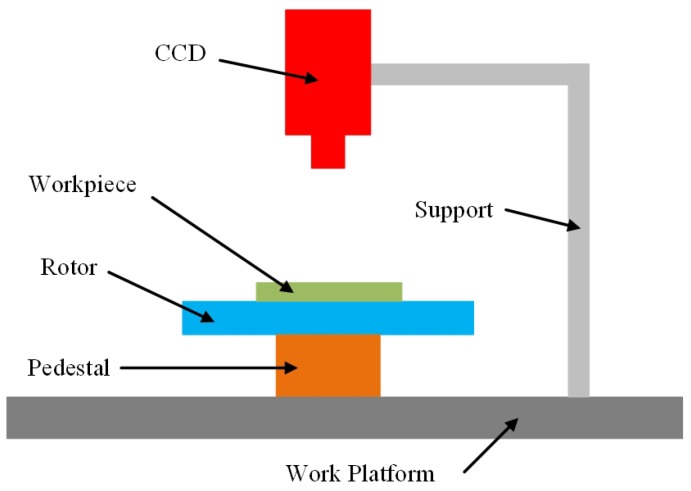
Schematic of the rotation-angle measurement assessment system.

A schematic of the rotation-angle measurement assessment system is shown in Figure 5. The experimental set up consists of a CCD, a computer, a support, and rotary equipment, which includes a pedestal and a rotor. A dial is fixed on the surface of the rotor, and the workpiece is placed on the dial. The workpiece is rotated by the rotor.

**Figure 6:**
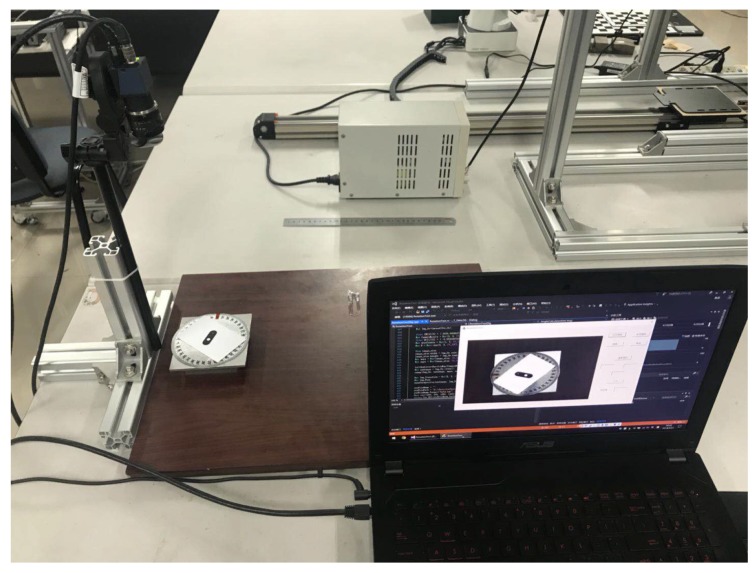
Experimental set up of the LIRS assessment system.

The CCD model is MER-200-14GC. It has a 12-mm lens, and its image resolution is 1628×1236. A support with three degrees of freedom is used to adjust the camera pose. For convenience, the camera optical axis is made perpendicular to the work plane by adjusting the support. A photograph of the experimental set up is shown in Figure 6.

The LIRS method is coded in C++ and compiled for 64 bits under Windows 10, and OpenCV 3.2 is used to process images. The program is executed using an Intel(R) Core(TM)i5-6300HQ CPU running at 2.30 GHz with 16 GB RAM. The workpiece is rotated from $1^{\circ }$ to $360^{\circ }$ in steps of $1^{\circ }$. One image is obtained per rotation, and the first image is regarded as the reference. Subsequently, rotation angles are calculated between the reference and the other images.

#### 3.3.1. Accuracy

**Figure 7:**
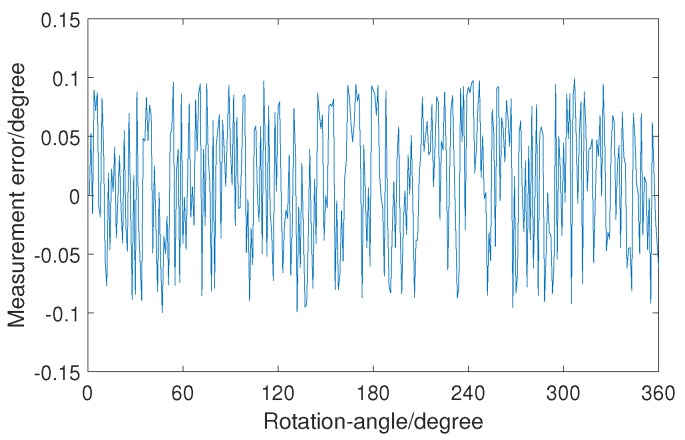
Measurement error of rotation angle in the experiment when the rotation angle is $1^{\circ }$–$360^{\circ }$.

The measurement error is shown in Figure 7. The maximum measurement error is less than $0.1^{\circ }$, which indicates that the LIRS method has a high accuracy. In other words, the LIRS method can be used to realize rotation-angle measurement with the whole angle range of $1^{\circ }$–$360^{\circ }$ in an automatic sorting system.

#### 3.3.2. Time Consumption

**Figure 8:**
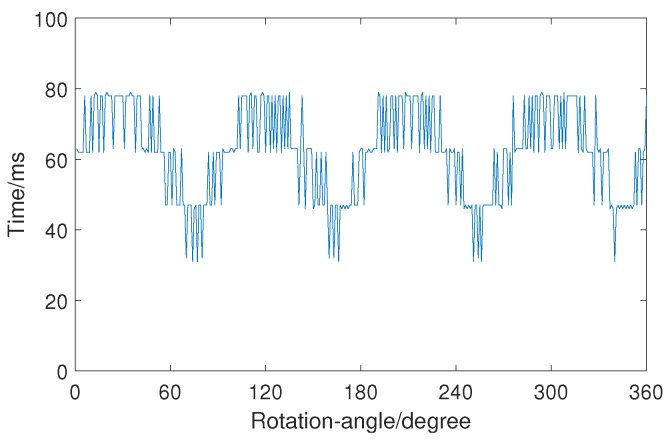
Time consumption of rotation-angle measurement when the rotation angle is $1^{\circ }$–$360^{\circ }$.

**Figure 9:**
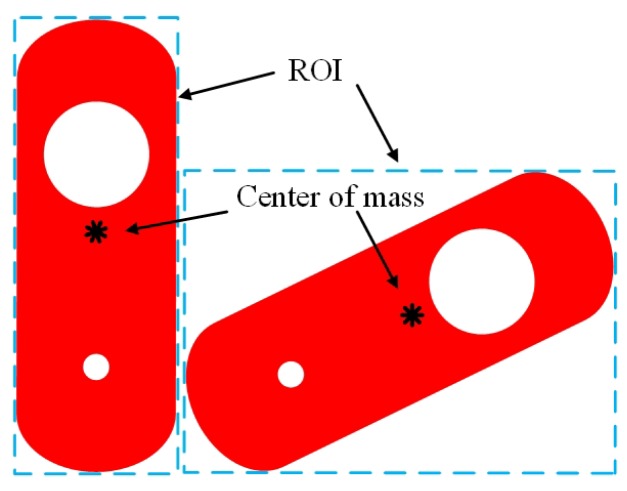
Schematic of ROI selection with the minimum external rectangle.

The time consumption of LIRS method is shown in Figure 8. The average time to calculate a rotation-angle is 62.1136 ms. The time-consumption curve shows large fluctuations because the images have different sizes. Each image shows the region of interest (ROI), which is determined based on the minimum external rectangle. The size of the ROI differs after rotation, as shown in Figure 9. Therefore, the time consumption shows large fluctuations when the whole angle range is measured.

There may be several workpieces in an image, and the execution of this program is sequential. Therefore, the time consumption is high. If the program is run in a field-programmable gate array (FPGA) device, the parallel-computing features of the FPGA device can be used to reduce the operating time substantially, further improving the efficiency of the LIRS method.

#### 3.3.3. Generality

The LIRS method is designed to overcome the limitation of rotation-angle measurement methods based on geometric moments. The LIRS method has an iteration number *n* and a deviation angle α, which can adjust the orientation of the separation line. The LIRS method can find a separation line for all non-centrosymmetric workpieces. This separation line will be determined uniquely after the deviation angle is selected. Therefore, the LIRS method has a higher flexibility and a better generality compared to the conventional method because it is suitable for workpieces of all shapes.

#### 3.3.4. Image Size

**Figure 10:**
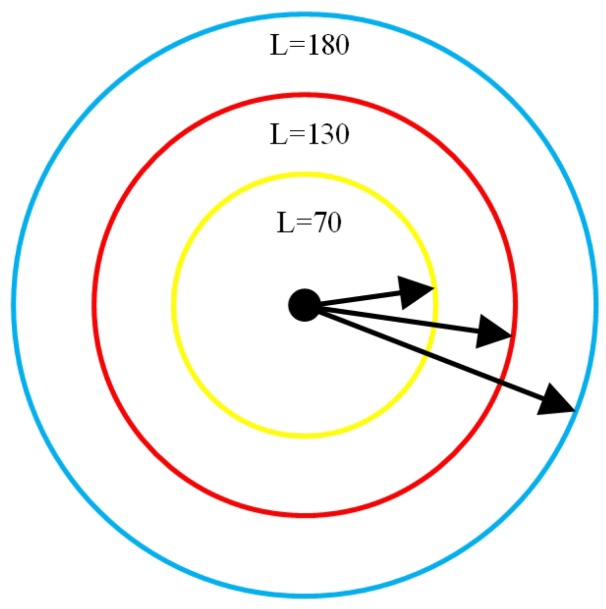
Schematic of the image size.

The relationship between the length of the direction vector and the measurement error should be consider since discretization error exist. Assume that the length of the direction vector is *l*. Take the starting point of the vector as the center and draw an one-pixel circle. The maximum directions which the vector could represent is equal to $A_{n}$, which is also the number of pixels on the circle. Therefore, with more pixels on the circle, the direction vector can represent more directions. As Figure 10 shows, L=70,130,180 are selected, and the maximum numbers of angles are $A_{n}=636,792,1312$, respectively.

The discretization error between the measured value and the actual value decreases when *l* is larger. As the number of direction increases, the discrete values are closer to being continuous values. Consequently, the accuracy of the LIRS method is increased. For the same workpiece, the length of the direction vector can be increased by selecting a suitable lens and reducing the distance between the camera and the workpiece. However, this will increase the size of the ROI and time consumption. It is necessary to obtain the optimal solution between time consumption and accuracy.

## 4. Rotation-Angle Measurement Model

### 4.1. Modeling

**Figure 11:**
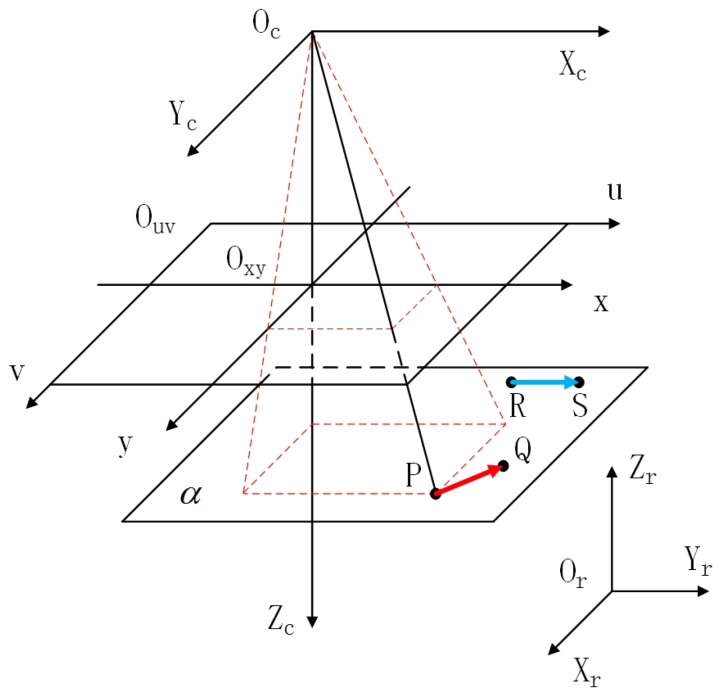
Geometry of the ideal camera model.

When the optical axis is non-perpendicular to the work plane, a dimensional measurement error will occur. In other words, the accuracy of dimensional measurement is affected by camera pose. However, the relationship between the accuracy of rotation-angle measurement and camera pose has not been studied. Therefore, a rotation-angle measurement model needs to be established. Figure 11 shows the basic geometry of the ideal camera model. Three steps are necessary because only an ideal camera model is addressed [26]. For convenience, the pose in which the optical axis is perpendicular to the work plane is called the ideal pose. All other camera poses are non-ideal.

There are four coordinate systems in this model. The camera coordinate system is composed of $X_{c}$, $Y_{c}$, and $Z_{c}$ axes and the point $O_{c}$. The robot coordinate system is treated as the world coordinate system, which is composed of $X_{r}$, $Y_{r}$, and $Z_{r}$ axes and the point $O_{r}$. The pixel coordinate system is composed of *u* and *v* axes and the point $O_{uv}$. The image coordinate system is composed of *x* and *y* axes and the point $O_{xy}$. The work plane is represented by α. For convenience, we assume that the $Z_{c}$ axis is perpendicular to the work plane, and the height of the workpiece is neglected. For any point *P* in α, its image coordinates can be expressed as Equations (13)–(17).

(13) $$Z_{c}\left[\begin{array}{c} u\\ v\\ 1 \end{array}\right]=\left[\begin{array}{ccc} f/dx & 0 & u_{0}\\ 0 & f/dy & v_{0}\\ 0 & 0 & 1 \end{array}\right]\left[\begin{array}{c} X_{c}\\ Y_{c}\\ Z_{c} \end{array}\right]$$

(14) $$\left[\begin{array}{c} X_{c}\\ Y_{c}\\ Z_{c} \end{array}\right]=R_{z}R_{y}R_{x}\left(\left[\begin{array}{c} X_{w}\\ Y_{w}\\ Z_{w} \end{array}\right]-\left[\begin{array}{c} t_{x}\\ t_{y}\\ t_{z} \end{array}\right]\right),$$

(15) $$R_{x}=\left[\begin{array}{ccc} \cos\alpha & -\sin\alpha & 0\\ \sin\alpha & \cos\alpha & 0\\ 0 & 0 & 1 \end{array}\right],$$

(16) $$R_{y}=\left[\begin{array}{ccc} 0 & 0 & 1\\ 0 & \cos\beta & -\sin\beta \\ 0 & \sin\beta & \cos\beta \end{array}\right],$$

(17) $$R_{z}=\left[\begin{array}{ccc} \cos\gamma & 0 & -\sin\gamma \\ 0 & 1 & 0\\ -\sin\gamma & 0 & \cos\gamma \end{array}\right],$$

where *f* is focal length, dx and dy are the distances between adjacent pixels in the *u* and *v* axes, respectively. $u_{0}$ and $v_{0}$ are row and column numbers of the center. ${\left[t_{x},t_{y},t_{z}\right]}^{\prime }$ is a translation vector from the robot coordinate to the camera coordinate system. $R_{x}$, $R_{y}$, and $R_{z}$ are three rotation matrixes, which are multiplied in the order of Equation (14). α, β, and γ are three angles. Equation (13) describes the relationship between camera coordinates system and pixel coordinates system. Equation (14) describes the relationship between robot coordinate system and camera coordinate system. The equation of coordinate transformation between pixel coordinate system and the robot coordinate system is established by using this two equations.

For convenience, vector $\vec{RS}=\left(1,0,0\right)$ is considered as the object pose, and the workpiece is abstracted as a vector $\vec{PQ}=\left(\Delta x,\Delta y,0\right)$. $\vec{RS}$ and $\vec{PQ}$ are represented by blue and red arrow in Figure 11, respectly. The work plane in robot coordinates is Zr=Z. The center of mass is treated as the starting point *P*, and the center of mass of region $D_{s}$ is regarded as the ending point *Q*. Therefore, the angle in the counterclockwise direction between $\vec{PQ}$ and $\vec{RS}$ can be regarded as the rotation angle.

The ideal value of the rotation angle is

(18) $$\theta_{i}^{\prime }=\arccos\frac{\vec{PQ}\times \vec{RS}}{\left|\vec{PQ}\right|\left|\vec{RS}\right|},$$

(19) $$\theta_{i}=f\left(\theta_{i}^{\prime }\right),$$

where *f* is an adjusting function that makes the value range of the rotation angle $\left[0^{\circ },360^{\circ }\right)$.

The measured value is obtained by substituting Equation (13) into Equation (18) and simplifying, as expressed by Equation (20)

(20) $$\theta_{r}^{\prime }=\arccos\frac{f_{n1}+t_{y}f_{n2}\left(1-c^{2}\right)+C\left(Af_{n2}-t_{y}k\sin y\right)}{0.5\sqrt{f_{d1}^{2}+f_{d2}^{2}}\sqrt{f_{d3}t_{y}^{2}+f_{d4}}},$$

(21) $$\theta_{r}=f\left(\theta_{r}^{\prime }\right),$$

where

(22) $$\left\{\begin{array}{c} f_{n1}=kA\sin\beta -d^{2}-\cos\alpha \\ f_{n2}=-t_{y}+kt_{x}+\frac{b}{t_{z}-Z}\\ f_{n3}=-2t_{y}+kt_{x}+\frac{b}{t_{z}-Z}\\ f_{d1}=k\cos\beta -Bf_{n2}-D\\ f_{d2}=\cos\alpha -f_{n2}\sin\alpha \\ f_{d3}=3-2\left(C^{2}-B^{2}\right)-\cos2\alpha \\ f_{d4}=3-2\left(A^{2}-D^{2}\right)+\cos2\alpha +8t_{y}AC\\ A=\sin\alpha \cos\beta \\ B=\cos\alpha \sin\beta \\ C=\cos\alpha \cos\beta \\ D=\sin\alpha \sin\beta \end{array}\right.$$

y=kx+b is a line corresponding to $\vec{PQ}$ in robot coordinates.

Thus, the rotation-angle measurement model has been established, and the difference between $\theta_{i}$ and $\theta_{r}$ is the rotation-angle measurement error.

### 4.2. Simulation and Discussion

It can be seen that the measured value is affected by several parameters, which can be divided into two categories. The first includes α, β, $t_{x}$, $t_{y}$, and the difference between the work plane and optical center $t_{z}-Z$. These six parameters will be confirmed after the camera is installed. There are only two angles in the model, and γ is not included. It can be seen that γ is uncorrelated with the measured value, and camera rotation around the its optic axis can be neglected in installation. Thus, camera-installation flexibility is improved in the automatic sorting system. The second category includes *k* and *b*. *k* is the tangent value of the rotation angle, and *b* is the position of the vector with the angle α. When the vector moves along the line, the measured value remains invariant. Otherwise, it will be changed. This means that different measured values would be obtained for some vectors that have the same rotation angle but dissimilar positions. This case would result in measurement error.

**Figure 12:**
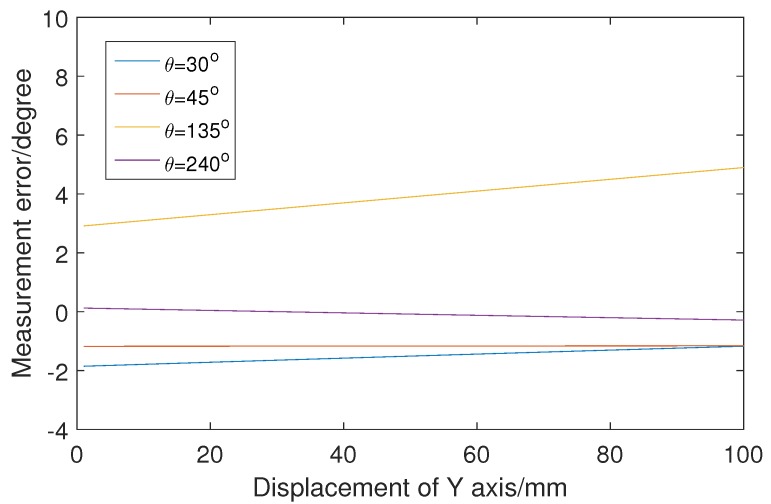
Measurement error in the simulation experiment when vectors move along the line y=50.

**Figure 13:**
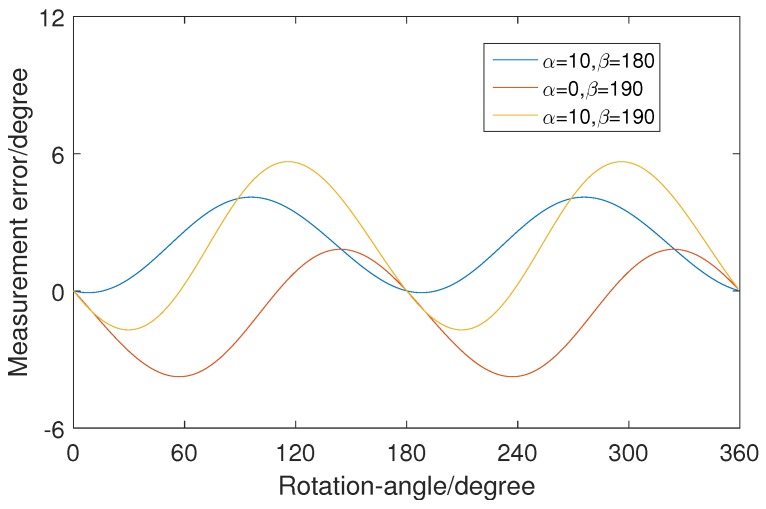
Measurement error in the simulation experiment when the vector rotates around its starting point.

Figure 12 shows curves of rotation-angle measurement error when four vectors move along the line y=50. An approximately linear relationship exists between displacement and measurement error. The polarity and the rate of error are related to the vectors. This means that different measured values would be obtained when the workpiece is located at different positions with the same rotation angle. Figure 13 shows the rotation-angle measurement error in simulation with four values of α and β. The vector rotates around its starting point in steps of $1^{\circ }$. For vectors with different values of α and β, the measurement-error curves are different. The measured values are different, when the same vector is selected with different values of α and β. That is, when the same workpiece is measured with different camera poses, the measured values are different. The polarity and value of the error is related to the camera pose.

To reduce the rotation-angle measurement error to zero, the following condition should be met:

(23) $$\theta_{i}-\theta_{r}=0.$$

Then, Equation (24) will obtained:

(24) α=0,β=0.

It can be seen that the measurement error is always present only if the camera is in a non-ideal pose.

### 4.3. Method for Correction of Rotation-Angle Measurement Error

To meet the condition of perpendicularity, camera should be adjusted by the support before the measurement. The rotation-angle measurement error will always exist when the camera is in a non-ideal pose, reducing the accuracy of rotation-angle measurement. To make the measured value accurate, it is necessary to keep the camera in the ideal pose. In other words, the optical axis needs to be adjusted to be perpendicular to the work plane. However, this condition cannot be met easily in industrial environments, because of camera-installation errors or position limitations. The actual pose could not be coinciding with the ideal pose completely. Therefore, the rotation-angle measurement error needs to be corrected.

When the camera is in a non-ideal pose, the $Z_{c}$ coordinate of a point on the work plane will be changed form a constant to a variable. The relationship between the image coordinates and camera coordinates can be expressed as follows:

(25) $$\Delta u=f\frac{X_{1}Z_{2}-X_{2}Z_{1}}{dxZ_{1}Z_{2}},\Delta v=f\frac{Y_{1}Z_{2}-Y_{2}Z_{1}}{dyZ_{1}Z_{2}},$$

where $\left(X_{1},Y_{1},Z_{1}\right)$ and $\left(X_{1},Y_{1},Z_{1}\right)$ are two camera coordinates in the work plane. dv and du are the differences of image coordinates. There is no linear relationship between $\sqrt{{\left(\Delta u\right)}^{2}+{\left(\Delta v\right)}^{2}}$ and $\sqrt{{\left(X_{1}-X_{2}\right)}^{2}+{\left(Y_{1}-Y_{2}\right)}^{2}}$. Therefore, the image will be distorted. This is the primary cause of the rotation-angle measurement error.

A rotation-angle error measurement correction (REMC) method with an error-correction matrix is presented to reduce the rotation-angle measurement error. A binary function ω is employed to multiply with $Z_{c}$ and keep the result constant. A linear relationship will be kept between the image coordinates and camera coordinates after mapping. The REMC method is illustrated in detail below.

A correction matrix *A* is introduced as follows:

(26) $$\left[\begin{array}{c} u^{\prime }\\ v^{\prime }\\ 1 \end{array}\right]=\frac{1}{\omega }\left[\begin{array}{ccc} a_{11} & a_{12} & a_{13}\\ a_{21} & a_{22} & a_{23}\\ a_{31} & a_{32} & a_{33} \end{array}\right]\left[\begin{array}{c} u\\ v\\ 1 \end{array}\right],$$

(27) $$\omega =ua_{31}+va_{32}+a_{33}.$$

The relationship between the image coordinate system $\left(u,v\right)$ and camera coordinate system $\left(X_{c},Y_{c},Z_{c}\right)$ can be expressed as follows:

(28) $$u=\frac{fX_{c}}{Z_{c}dx}+u_{0},u=\frac{fY_{c}}{Z_{c}dy}+v_{0}.$$

Then, Equation (13) can be rewritten as follows:

(29) $$Z_{c}\omega \left[\begin{array}{c} u^{\prime }\\ v^{\prime }\\ 1 \end{array}\right]=\left[\begin{array}{ccc} F_{11}^{\prime } & F_{12}^{\prime } & F_{13}^{\prime }\\ F_{21}^{\prime } & F_{22}^{\prime } & F_{23}^{\prime }\\ F_{31}^{\prime } & F_{32}^{\prime } & F_{33}^{\prime } \end{array}\right]\left(\left[\begin{array}{c} X_{w}\\ Y_{w}\\ Z_{w} \end{array}\right]-\left[\begin{array}{c} t_{x}\\ t_{y}\\ t_{z} \end{array}\right]\right),$$

where *F* is a coefficient matrix and $Z_{c}\omega$ can be expressed as

(30) $$Z_{c}\omega =a_{31}f_{x}X_{c}+a_{32}f_{y}Y_{c}+s\left(a_{31}u_{0}+a_{32}v_{0}+a_{33}\right).$$

The work plane in the camera coordinate system can be expressed as follows:

(31) $$aX_{c}+bY_{c}+cZ_{c}-A=0,$$

where *a*, *b*, *c*, and *A* are constant parameters. Three parameters $a_{31}$, $a_{32}$, and $a_{33}$ must exist to ensure the equation holds:

(32) $$Z_{c}w=A.$$

Then, $\left(u^{\prime },v^{\prime },1\right)$ can be obtained as follows:

(33) $$\left[\begin{array}{c} u^{\prime }\\ v^{\prime }\\ 1 \end{array}\right]=\frac{1}{A}\left[\begin{array}{ccc} F_{11}^{\prime } & F_{12}^{\prime } & F_{13}^{\prime }\\ F_{21}^{\prime } & F_{22}^{\prime } & F_{23}^{\prime }\\ F_{31}^{\prime } & F_{32}^{\prime } & F_{33}^{\prime } \end{array}\right]\left(\left[\begin{array}{c} X_{w}\\ Y_{w}\\ Z_{w} \end{array}\right]-\left[\begin{array}{c} t_{x}\\ t_{y}\\ t_{z} \end{array}\right]\right).$$

*A* is the $Z_{c}$ value of the work plane in the camera coordinate system. It can be seen that the $Z_{c}$ value can remain invariant during the mapping process. Therefore, the measurement error caused by camera pose will be reduced when $\left(u^{\prime },v^{\prime }\right)$ is used to calculate the rotation angle.

The experimental system is shown in Figure 6. The optical axis is adjusted using the support to be non-perpendicular to the work plane, and the obtained results are listed in Table 1. It can be seen that the REMC method can reduce the rotation-angle measurement error caused by a non-ideal camera pose, and the error is less than $0.1^{\circ }$. Therefore, the proposed method is effective and meets the requirements.

The correction matrix is selected as follows:

(34) $$A=\left[\begin{array}{ccc} 4.5985 & 0.0779219 & -1904.15\\ 0.0572827 & 4.58486 & -2660.6\\ 2.07701\times 10^{-5} & 4.75542\times 10^{-5} & 1 \end{array}\right].$$

## 5. Experiment

**Figure 14:**
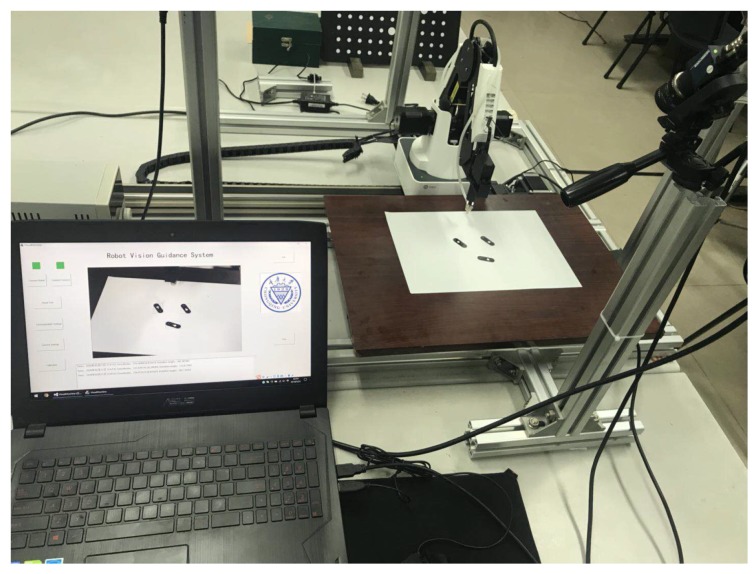
Automatic sorting system with machine vision.

An automatic sorting system with machine vision is established, as shown in Figure 14. A robot (Dobot Magician) with a four degrees of freedom robot is used in this system. Four stepping motors are used to drive a manipulator, which moves with a re-orientation accuracy of 0.2 mm. The software is coded by MFC with OpenCV 3.2 and consisted of three parts: (1) a camera and a robot control system including initialization, start and stop functions, and parameter setting; (2) a real-time display system consisting of an image display and information display; and (3) an information storage system designed to save important data during program operation. The correction matrix *A* is selected as follows:

(35) $$A=\left[\begin{array}{ccc} 1.45457 & 0.383242 & -902.439\\ -0.220291 & 1.86919 & -318.915\\ 1.57966\times 10^{-6} & 0.000271224 & 1 \end{array}\right].$$

**Figure 15:**
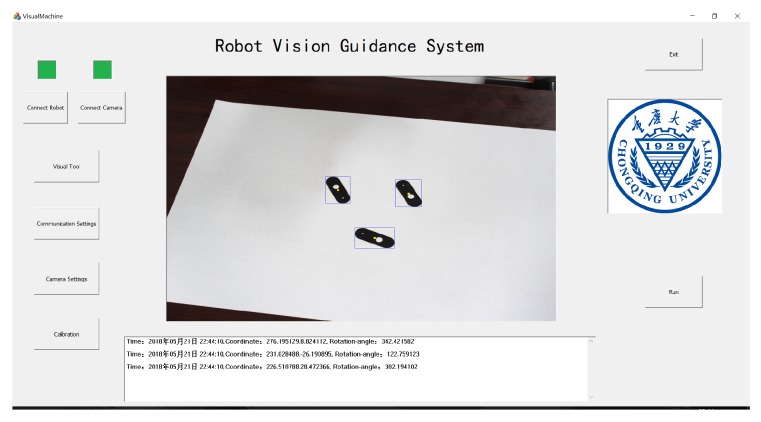
Result of the experiment.

The workpiece is a uniform-thickness thin sheet with two holes of different diameters. The experimental result is shown in Figure 15. The image with the blue external rectangle and yellow point is shown on the main interface. Key information is shown in the message region. The results show that the rotation angles are obtained accurately, and the workpieces could be placed correctly by this system. Therefore, the LIRS and REMC method could be used in automatic sorting systems in industrial environments.

## 6. Conclusions

The rotation angle is an important parameter in an automatic sorting system. To accurately measure the rotation angles of plane workpieces for an automatic sorting system, the LIRS method was proposed. This method overcomes limitation of the conventional method based on geometric moments, and it is suitable for workpieces of all shapes. Experimental results show that the measurement error of the LIRS method is less than $0.1^{\circ }$, and the measurement range is between $0^{\circ }$ and $360^{\circ }$. Therefore, the LIRS method meets the requirements of automatic sorting in industrial environments. However, the average measurement time is approximately 62.1136 ms, which leaves much room for improvement.

A model was established for studying the relationship between camera pose and rotation-angle measurement error. Then, a formula for calculating the error was derived. The simulation results show that the measurement error will always exist when the camera is in a non-ideal pose. The value and polarity of the measurement error are related to the camera pose and location of the workpiece. Subsequently, the REMC method was designed to correct the rotation-angle measurement error. The experimental results show that the REMC method is effective, and the measurement error with the REMC method is less than $0.12^{\circ }$.

Finally, an automatic sorting system with the LIRS and REMC method was established, and sorting experiments were conducted. The two proposed methods yielded accurate rotation angles, and plane workpieces could be placed correctly by this system.

## Figures and Tables

**Table 1:** Experimental results obtained when the REMC method is employed in the experiment under a non-ideal camera pose.

Ideal Value	Measured Value	Correction Value	Error
$30^{\circ }$	$31.15^{\circ }$	$30.11^{\circ }$	$0.11^{\circ }$
$60^{\circ }$	$62.95^{\circ }$	$60.06^{\circ }$	$0.06^{\circ }$
$120^{\circ }$	$121.97^{\circ }$	$119.04^{\circ }$	$0.04^{\circ }$
$150^{\circ }$	$150.31.32^{\circ }$	$150.03^{\circ }$	$0.03^{\circ }$
$210^{\circ }$	$210.21^{\circ }$	$209.09^{\circ }$	$0.09^{\circ }$
$240^{\circ }$	$242.61^{\circ }$	$240.05^{\circ }$	$0.05^{\circ }$
$310^{\circ }$	$311.73^{\circ }$	$319.03^{\circ }$	$0.03^{\circ }$
$330^{\circ }$	$330.82^{\circ }$	$330.06^{\circ }$	$0.06^{\circ }$
